# Supplementary material for: Stacking Model‐Based Classifiers for Dealing With Multiple Sets of Noisy Labels
Source: Biom J. 2025 Mar 12;67(2):e70042. doi: 10.1002/bimj.70042 (PMC11898607; doi:10.1002/bimj.70042)
Supplement: Supplementary file 1 — Supporting Information [file BIMJ-67-e70042-s001.zip › density-based-ensemble-model-main/competitors_real_data/netlab-master/netlab-master/help/demgtm1.htm]

Netlab Reference Manual demgtm1


# demgtm1

## Purpose

Demonstrate EM for GTM.

## Synopsis

```
demgtm1
```

## Description

This script demonstrates the use of the EM
algorithm to fit a one-dimensional GTM to a two-dimensional set of data
using maximum likelihood. The location and spread of the Gaussian kernels
in the data space is shown during training.

## See Also

`demgtm2`, `gtm`, `gtmem`, `gtmpost`

---

**Pages:**
Index


---

Copyright (c) Ian T Nabney (1996-9)
